# TC-99m HMPAO Brain Blood Flow Imaging in the Dementias with Histopathologic Correlation in 73 Patients

**DOI:** 10.1155/2011/409101

**Published:** 2010-12-01

**Authors:** Frederick J. Bonte, Linda Hynan, Thomas S. Harris, Charles L. White

**Affiliations:** ^1^Department of Radiology, Nuclear Medicine Center, The University of Texas Southwestern Medical Center at Dallas, 5323 Harry Hines Boulevard, Dallas, TX 75390, USA; ^2^Department of Clinical Sciences, The University of Texas Southwestern Medical Center at Dallas, 5323 Harry Hines Boulevard, Dallas, TX 75390, USA; ^3^Division of Neuropathology, Department of Pathology, The University of Texas Southwestern Medical Center at Dallas, 5323 Harry Hines Blvd., Dallas, TX 75390, USA

## Abstract

The purpose of this study is to determine the value of Tc-99m HMPAO SPECT in the diagnosis of the dementias. Tc-99m HMPAO was used with a 3-camera scanner to produce 5 sets of sectional images of the brain. Images were further processed using Statistical Parametric Mapping. Diagnosis was made by a physician blinded to the clinical diagnosis. Results in 73 subjects were compared with a neuropathologic study of the brain at autopsy. Data were analyzed for sensitivity, specificity, positive and negative predictive values and accuracy. These results are compared with several other studies performed with Tc-99m HMPAO SPECT with histopathologic correlation. This procedure is widely available and relatively inexpensive and may be of value in patients with dementias and problematic diagnoses. Further, a degree of differential diagnosis between Alzheimer's and Frontotemporal diseases may be effected. The study was approved by our Institutional Review Board.

## 1. Introduction

This is one of a continuing series [3–7] of reports on a group of patients studied with Tc-99m hexamethylpropyleneamine oxime (HMPAO, Mallinckrodt, Inc.) SPECT brain blood flow. This paper will add 24 patients to the previously described series and will constitute the final report on this imaging study.

## 2. Materials and Methods

**Figure 1:**
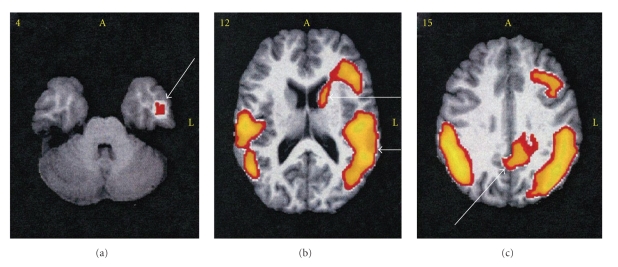
The patient was a 64-year-old male. An SPECT scan was performed as described and further processed with SPM [1, 2]. The areas of interest displayed in yellow represent groups of voxels within which regional blood flow was below normal; *P* is ≤.001. Three transaxial sections were selected from a group of 24. Image (a) shows reduced flow in a small volume of the left hippocampus (→). (b) ↑ shows reduced RCBF in the left caudate and adjacent lateral frontal cortex (↑). The lower arrow indicates reduced flow in the left posterior temporal and occipital regions. (c) There is reduced RCBF in the posterior cingulate cortex (↑). Left frontal and bilateral parietal regions of low flow are also noted.

SPECT was carried out with a three-camera scanner (PRISM Picker 3000, Cleveland, OH) and a tracer dose of 740 MBq of Tc-99m HMPAO. After intravenous administration, a period of 90 m was allowed for distribution of the agent. The patient then was asked to recline on a couch with comfortable bracing of the head within the detection space of the scanner. Data were acquired with high-resolution fan-beam collimators in a 128 × 128 matrix. Data sets were acquired at 3° intervals for 30 s each, to a total of 40 sets. Correction for fan-beam collimator geometry was made during acquisition. Data were reconstructed as 12.5-cm-radius cylinders. Data were then multiplied by a factor of 4 and processed with a Ramp filter, with a pixel size of 1.89 mm. Attenuation correction was applied. Postfiltering was carried out with a three-dimensional pass filter with an upper limit of 10 and a cutoff of 2.49. With use of a pixel size of 5–7 mm, contiguous transaxial sections were generated parallel to the canthomeatal line, as well as coronal and sagittal views. Two special temporal sections were also generated. Initially, scans were displayed in a color scale of the “heated body” type, but the final 49/73 cases were further processed using Statistical Parametric Mapping [1, 2]. Data were reoriented parallel to a plane passing through the anterior and posterior commissures and were sectioned to produce the five views described above. SPM patient image data were then compared with the summed images of normal 20 volunteers for interpretation (see Figure 1).

An example of the application of this method may be seen in the case represented by Figure 1. As usual, the clinical state of the patient was unknown to the interpreter of the study images. The 3 images are transaxial sections selected from the study of a 64-year-old male performed as described above. The SPECT data were then processed by SPM and displayed so as to show brain volumes within which regional cerebral blood flow (RCBF) was reduced below the value derived from the group of 20 normal volunteers in a suitable age group, with a statistical significance of *P* being ≤.001. The findings of reduced RCBF in the hippocampus, temporal, and parietal lobes, together with the important finding of flow reduction in the posterior cingulate cortex, a finding seen almost exclusively with AD, and reduced RCBF is also seen in the left caudate nucleus, and in the inferior occipital cortex (→B). The latter distribution suggests the possibility of a component of Lewy body disease. Pathologic findings supported the scan diagnosis of AD with possible Parkinson's disease, with many Lewy bodies present.

The interval between scan and autopsy was from 1 m to 181 m (mean = 71.1 m)

## 3. Results and Discussion

The results of this series are summarized in Table 1 in terms of the diagnosis of AD, with or without Lewy bodies. In terms of correlation with histopathology, SPECT findings are given as true positive, true negative, false positive and false negative.

Table 2 shows statistical evaluation of the findings in Table 1 in terms of proportion, with 95% confidence intervals, for sensitivity, specificity, positive predictive value, negative predictive value and accuracy.

In the latter part of this study, an effort was made to evaluate the possibility of a diagnostic track for Frontotemporal disease. Results suggested the possibility of such a track, but an insufficient number of cases in this study prevent valid statistical evaluation. In any case, findings in the Frontotemporal group would probably not permit differential diagnosis among the various entities.

The results of this study were compared with those of 2 previous studies which were performed with Tc-99m HMPAO SPECT, with autopsy confirmation [8–10]. In these studies, accuracy in the detection of AD was approximately 80%. Accuracy in the present study is approximately 90%. The apparent improvement is probably due to increasing quality of SPECT instruments together with scan data treatment with Statistical Parametric Mapping. This procedure facilitates the identification of brain volumes in which significant reduction of RCBF has occurred. It is especially helpful in the detection of flow reduction in the posterior cingulate cortex [11], which is a strong marker for AD, when present [7]. SPECT brain imaging in the dementias remains a relatively inexpensive procedure, compared with the several applications of PET, and would probably be a satisfactory substitute when PET with one of the newer agents is unavailable. Again, however, the small size of our patient group does not permit us to make statistical valid conclusions relative to the value of SPECT.

## 4. Conclusions

SPECT brain blood flow imaging has the ability to detect active Alzheimer's disease with approximately 90% accuracy. It can be postulated that this technique is capable of differential diagnosis between AD and the Frontotemporal dementias. Further, interpretation of the scan data may be enhanced by a treatment such as Statistical Parametric Mapping. However, we do not believe that the number of patients in our study group permits us to claim statistical significance. Further, the considerable variation in time elapsed between scan and autopsy among the patient group does not permit us to evaluate the role of this study in the detection of early disease.

##  Conflict of Interests

The authors have no conflict of interests in this work.

## Figures and Tables

**Table 1:** Diagnosis of AD with or without Lewy body disease.

True positive	44
True negative	22
False positive	4
False negative	3

**Table 2:** For the diagnosis of AD with or without Lewy bodies.

Proportion	95% Confidence interval	
	Lower limit	Upper limit
Sensitivity: 44/47 = 93.62%	81.44%	98.34%
Specificity: 22/26 = 84.62%	64.27%	94.95%
Positive predictive value: 44/48 = 91.67%	79.13%	97.30%
Negative predictive value: 22/25 = 88.00%	67.66%	96.85%
Accuracy: 66/73 = 90.41%	80.67%	95.73%
